# Rights and Liabilities of Dentists at Common Law

**Published:** 1882-07

**Authors:** Jas. G. Dunning

**Affiliations:** The Springfield Bar.


### ARTICLE III.

### *Rights and Liabilities of Dentists at Common Law.*

BY JAS. G. DUNNING, L. L. B., OF THE SPRINGFIELD BAR.

The rights and liabilities of dentists in the practice of their profession are to be determined by the same rules that have been laid down for physicians and surgeons, and business experts in general.

A dentist is obliged in each case to apply such diligence as good dentists, acting under similar circumstances, would apply. The simple question is, did he in the particular case exhibit such skill and diligence as good dentists in such cases are accustomed to exhibit? When a man offers himself to the public as a dentist, the law requires that he be possessed of that reasonable degree of learning and skill which is ordinarily possessed by others of his profession who are in good standing as to qualifications. This rule does not require that he should have the highest skill, or largest experience, or most thorough education, equal to the most eminent of the profession of the whole country, but it *does* require that he should not when uneducated, ignorant and unfitted, palm himself off as a professional man, well qualified, and go on blindly in the duties of the profession.

But a dentist, qualified within this rule *may* be guilty of negligence and malpractice. The law requires and implies, as a part of the contract, that when a dentist undertakes professional charge of a patient, he will use reasonable and ordinary care and diligence in the case. The law implies that he agrees to use his best skill and judgment, but not that he shall possess the best skill and the best judgment. There is no doubt of the legal right of a dentist to refuse to take charge of a particular case. When in charge, however, he is liable for any negligence, whether of omission, or commission, which produces injury to his patient. But a dentist who is acting gratuitously, is liable only for gross negligence. An inexperienced volunteer, who acts when no expert can be obtained, is liable only for gross negligence. But if, by forcing himself into the case, he thereby excludes a competent dentist, his liability is the same as that of such competent dentist. If the practitioner, however, frankly informs his patient of his want of skill, or the patient in some other way is fully aware of it, the latter cannot complain of the lack of that which he knew did not exist. But no recovery can be had in any case where there has been no injury. The implied liability of a dentist retained to treat any particular case extends no further, in the absence of any special agreement, than that he will indemnify his patient against any injurious consequences resulting from his want of proper degree of skill, care, or diligence in the execution of his employment. And in an action against a dentist for negligence, or malpractice, the plantiff, if he shows no injury resulting from negligence, or the want of due skill in the defendant, will not be entitled even to nominal damages.

If a patient, refusing to adopt the advice and remedies of his dentist, or to follow his directions in any way, thereby frustrates the latter's endeavors, or if he aggravates the case by his misconduct, he cannot charge to the dentist the consequences due to himself. It is the duty of the patient to co-operate with his dentist; and if he will not, or under the pressure of pain cannot, his neglect is his own misfortune.

The rule has been stated to be that a dentist is bound only to exercise ordinary skill and diligence, the *average* of that possessed by the profession as a body, and not of the thoroughly educated only; that is, that he will satisfy the law's requirements if he possess the "average capacity" of the members of his profession. But the true rule seems to be not what the *average* of the profession would do, but what an intelligent, responsible and respectable member of the profession would do under the same circumstances. The average skill of the profession, taking in good and bad, young and old, as a mass, is hard to reach; and if we count into the aggregate the young who have had no practice, and the old who have retired from practice, the average would give a standard lower than that which should be required. Nor is this the only reason why the test of the "average capacity" is inadequate. In a city there are many means of professional culture inaccessible to the country. In a city new books and appliances can be promptly purchased, and libraries easily visited. In a city also exists that intercourse with prominent professional men which leads not only to keenness and culture, but to the free interchange of new modes of treatment. In the country such opportunities do not exist. What is therefore due diligence and skill in the country is not due diligence and skill in the city: and what is due diligence and skill in the city is not due diligence and skill in the country. Hence, the question of diligence and skill in each particular case is to be determined, not by inquiring what would be the average diligence of the profession, but what would be the diligence of an honest, intelligent, responsible member of the profession in the position in which the defendant was placed.

The following case is reported in the 39th volume of the Maine Reports: Assumpsit for a ful set of artificial teeth for the defendant's wife. The contract was made with the wife with the knowledge and consent of defendant. When put into her mouth, she complained that they felt odd and pained her. The plate was then somewhat filed, but she still complained and declined to pay for them. It was agreed that she might take them away and return them on the following Monday, when she returned them and said she knew she never could wear them. Something further was done to the teeth, but she declined to pay for them and left them, although the plantiff forbade her doing so, and claimed his pay. There was conflicting evidence as to whether the teeth fitted her mouth. By one it was testified that they were a good piece of work, by another that they were a fair average piece of work, and by a third that they were nothing extra. Among other instructions the jury were told that "if the plaintiff had used all the knowledge and skill to which the art had at that time advanced, that would be all required of him." Verdict was for the defendant. Exceptions were taken to the above instructions by plaintiff's counsel, on the ground that they were misleading to the jury as implying that any less degree of skill would not satisfy the requirements of the law, and the full court held the above instruction erroneous as tending to hold the standard of professional requirements too high, and a new trial was ordered. In considering this case, the court said: "The highest degree of skill is not to be expected unless stipulated for; nor can it be reasonably required of all. If a dentist has used all the knowledge and skill to which his art had at the time advanced, no doubt that would be all required of him. But could so much be required of him? If it could, then every professional man would be bound to possess the highest attainments, and to excercise the greatest skill in his profession. Such a requirement would be unreasonable."

A plaintiff in an action against a dentist for malpractice must prove that the defendant assumed the character and undertook to act as a dentist without the education, knowledge and skill which entitled him to act in that capacity, or, he is bound to prove that having such education, knowledge and skill, he neglected to apply them with such care and diligence as in his judgment, properly exercised, the case must have appeared to require; in other words, that he neglected the proper treatment from inattention and carelessness.—*New England Journal of Dentistry.*

